# Conservation choice on the rare endangered plants *Glehnia littoralis*

**DOI:** 10.1093/conphys/coy002

**Published:** 2018-01-29

**Authors:** Yanxia Pan, Jianmin Chu, Hongxiao Yang

**Affiliations:** 1 Qingdao Agricultural University, Changcheng Road, Chengyang, Qingdao, Shandong Province 266109, China; 2 Key Laboratory of Tree Breeding and Cultivation, State Forestry Administration, Research Institute of Forestry, Chinese Academy of Forestry, Beijing 100091, China; 3 Qingdao Engineering Research Center for Rural Environment, Chengyang, Qingdao, Shandong Province 266109, China

**Keywords:** Coastal plant, conservation, domestication, functional decline, *Glehnia littoralis*, seed

## Abstract

The coastal herbs *Glehnia littoralis* have been domesticated as traditional medicines for many centuries. The domestication may have caused changes or declines of cultivated *G. littoralis* (CGL) relative to wild *G. littoralis* (WGL). By comparing fruit properties of CGL and WGL, we tested the hypothesis that domesticated *G. littoralis* have suffered major declines, and human cultivation cannot be sufficient to conserve this species. We collected fruits of CGL and WGL in the Shandong peninsula, China, and compared their buoyancy in seawater, germination potential after seawater immersion, and thousand-grain weights. Float rates of the WGL and CGL fruits were 95.6 (mean) ± 2.6% (standard deviation) and 30.0 ± 7.1%, respectively. The germination potential of CGL was significantly reduced, although the thousand-grain weights of CGL (21.85 ± 0.17 g) were higher than those of the WGL fruits (14.73 ± 0.21 g). These results suggest that the CGL have experienced significant declines relative to the WGL, presumably due to the loss of seawater inundation, selection and dispersal. These declines disfavour the persistence of CGL, and human domestication and cultivation are believed to be insufficient for conserving *G. littoralis*. Sand coasts where WGL still persists should be designated timely as nature reserves to conserve this species.

## Teaser

Inland cultivation caused declines of buoyancy and germination ability of *Glehnia littoralis* fruits, because of long-term dispersal failure. Inland transplanting is, therefore, insufficient for conserving this species. As a coastal specialist, *G. littoralis* can only persist for long along sand coasts, where storm surge is a natural vector for dispersal.

## Introduction

Domesticated plants provide humans with basic necessities, such as cereals, vegetables and herbal medicines. Humans have been making efforts to increase yields of these plants. However, their attempts may have also undermined the potentials for them to self-persist (Pujol *et al.*, 2005; Abbo *et al.*, 2014). Once these plants become extinct or expired, humans have to suffer the loss of precious resources or necessities. These domesticated plants must be conserved, and their wild relatives as useful candidates also need conservation (Pujol *et al.*, 2005; Khoury *et al.*, 2015; Cooke *et al.*, 2017).

*Glehnia littoralis* Fr. Schmidt ex Miq. is a rare species of herbs that is naturally distributed along sandy coasts of the North Pacific Ocean (Qian and Klinka, 1998; Hiraoka *et al.*, 2002; Kim *et al.*, 2006). They are critically endangered (CR) as IUCN estimated (http://rep.iplant.cn/prot/Glehnia%20littoralis). Their roots are traditional medicine to treat lung diseases and cancers (Matsuura *et al.*, 1996; Yoon *et al.*, 2010). To achieve a steady yield, Chinese people began to cultivate the herbs many centuries ago. The Shandong Peninsula is the centre for cultivating the species. The cultivated *G. littoralis* (CGL) were domesticated at least from the Ming Dynasty, by wild *G. littoralis* (WGL) ever along the local seashore, and some prevailing breeds have come into being. WGL used to thrive at most sandy coasts but is now endangered because of heavy tourism and other human disturbances (Kim *et al.*, 2005; Yang *et al.*, 2017).

Past studies on this species focused on medicine usage and cultivation (Hiraoka *et al.*, 2002; Xu *et al.*, 2010; Yoon *et al.*, 2010; Feng *et al.*, 2014; Sun *et al.*, 2015; Hee *et al.*, 2017). After it was pronounced as a rare endangered species, concerns on its living status and ecology increased. Its wild breeds were observed to adapt to germination and dispersal along temperate sand seashore (Tada *et al.*, 2013; Lee *et al.*, 2014; Zhang and Yang, 2015; Yang *et al.*, 2017). All the CGL are cultivated and dominated by humans, seriously departing from their normal life, thus, they may have changed or declined relative to WGL. With the cultivated and wild *G. littoralis*, we tested the hypothesis that domesticated *G. littoralis* may have suffered functional declines that can undermine their potentials for self-persistence, and human cultivation alone cannot be sufficient to conserve this species for long. To fulfil this, we compared abilities of the CGL and WGL fruits to disperse by seawater and to germinate after seawater dispersal.

## Materials and methods

### Materials

**Figure1:**
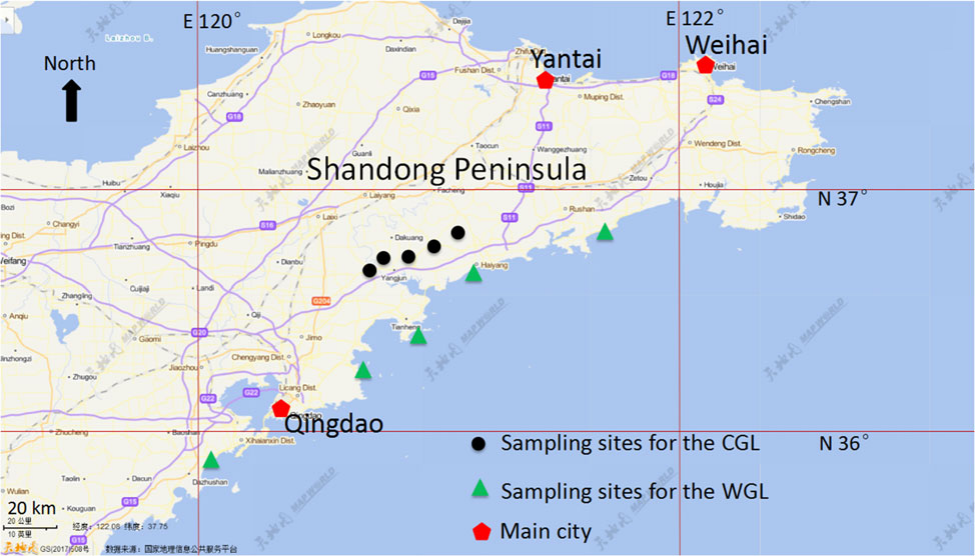
Sampling sites in the Shandong Peninsula, China.

We collected mature fruits from CGL and WGL in September 2014 in the Shandong Peninsula (Fig. 1). The CGL were grown by the locals on riparian sandy area. The WGL naturally grow at sandy coasts, and are subjected to occasional seawater inundation from extreme storm surges. CGL fruits were sampled from five sites, and WGL fruits were collected from five sandy coasts. The seeds were not unwrapped from the fruits and were maintained in natural states. The fruits were air-dried and stored. In addition, we collected sand from riparian area, which is suitable for this species, and placed it in plastic boxes in sizes of 40 cm (length) × 20 cm (width) × 10 cm (height) and with small drainage holes at box bases.

### Experiments

Buoyancy of the fruits in seawater and germination potential after seawater immersion are key adaptations to seawater dispersal (Nilsson *et al.*, 2010; Yang *et al.*, 2017). Without the buoyancy, the fruits would be selected against by seawater inundation and sink to the seabed, thus, suffering dispersal failure (Nilsson *et al.*, 2010; Yang *et al.*, 2012). Without the germination potential, they could not germinate even after they returned to sandy coasts with seawater (Yang *et al.*, 2017). Either of the cases is deadly for *G. littoralis* to survive and persist along sandy coasts. In this context, we designed the following experiments to compare buoyancy of the fruits in seawater and their germination potential after seawater immersion.

The first experiment was initiated in October 2014. We immersed 50 (grains) × 5 (samples) fruits of the CGL and 50 (grains) × 5 (samples) fruits of the WGL in seawater. The seawater was collected from the local sea and placed into 10 glass beakers, which were sufficiently large to allow each fruit to be in full contact with the water surface. One beaker corresponded to one sample, which contained 50 fruits. For 60 days, we observed how many grains remained afloat. We replaced the seawater in the beakers every 10 days, and a sieve with small holes was used to keep the fruits in the beakers.

Another experiment was also done. Every 10 days, we immersed one batch of fruits (CGL: 30 grains × 5 samples; WGL: 30 grains × 5 samples) in seawater. We replaced the seawater for the immersed fruits every 10 days. Thus, we prepared a total of seven batches with the immersion duration of 60, 50, 40, 30, 20, 10 and 0 days. After 2 months, we took away the fruits and sowed them 4 cm deep in the box with sand. In terms of our experiences, the depth of 4 cm is appropriate for germination. A total of 10 boxes were used for each batch: five boxes for the CGL and five boxes for the WGL. Each box contained 30 grains. The boxes were then placed outdoors and covered with a coarse-eyed mesh 2 m above to protect them from bird destruction. The seeds of the fruits germinated after winter. Upon germinating, we counted new seedlings every several days.

Thirdly, we measured thousand-grain weights of the air-dried fruits, five samples for the CGL and five samples for the WGL. By referring to this, we further assessed whether the fruit weights set back the seed germination.

### Statistical analyses

From the first experiment, we obtained float rates of the fruits for each sample. The float rate is defined as the number of floating fruits up to a certain day divided by the number of initially immersed fruits. Using a different-variance *t*-test, we examined whether the final float rates differed between the CGL and WGL.

From the second experiment, we derived germination indices (GI) of the fruits as follows: $GI=\overset{60}{\underset{t=1}{\sum }}\left(G_{t}/t\right)$, where *t* marked the *t*-th day in the 60 days of germination period, and *G_t_* is the number of newly emergent seedlings on the *t*-th day divided by the number of initially sown fruits, one fruit (one half naturally split from a schizocarp fruit) containing one seed (He *et al.*, 2008; Liu *et al.*, 2014). Using two-way ANOVA, we examined whether the GI values were affected by the sources of the CGL and WGL and the immersion duration (0, 10,……., 60 days).

We also examined whether thousand-grain weights of the fruits differed between the CGL and WGL using a different-variance *t*-test. The statistical analyses were performed using Origin 8.5 software (OriginLab, Northampton, USA).

## Results

**Figure 2:**
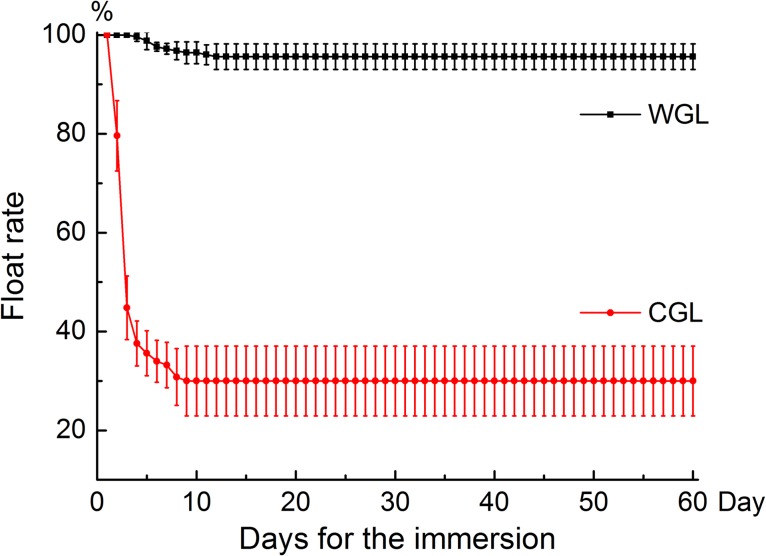
Buoyancy of *Glehnia littoralis* fruits in seawater (mean float rates ± standard deviation). WGL, wild *G. littoralis* from natural sandy coasts; CGL, cultivated *G. littoralis* from riparian sandy areas.

The float rates after 60-day seawater immersion for the WGL and CGL fruits were 95.6 (mean) ± 2.6% (standard deviation) and 30.0 ± 7.1%, respectively (Fig. 2). The *t*-test indicates that the float rates of the WGL fruits were significantly different from those of the CGL (*t*= 19.46, d.f. = 5.07, *P* < 0.001). This suggests that the domestication has significantly reduced buoyancy of the CGL fruits.

**Figure 3:**
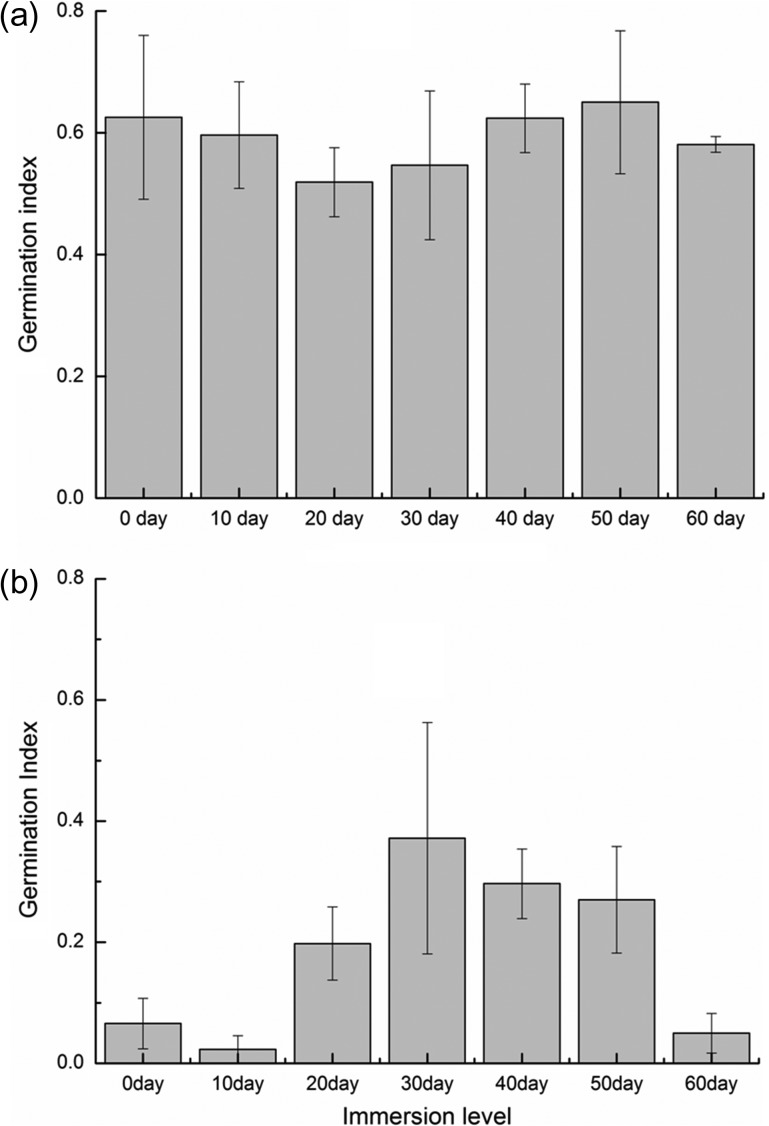
Effects of seawater immersion on the germination potential of *Glehnia littoralis*. (**a**) Wild *G. littoralis* from natural sandy coasts and (**b**) cultivated *G. littoralis* from riparian sandy areas.

The two-way ANOVA indicates that the *GI* of the fruits differed between the WGL and CGL (*F*_1,56_ = 356.17, *P* < 0.001) and also varied with immersion duration (*F*_6,56_ = 6.80, *P* < 0.001). This suggests that germination potential of the CGL fruits was significantly reduced relative to that of the WGL fruits (Fig. 3). Moreover, fruits of the CGL that did not experience seawater immersion presented much lower GI than those of the WGL with the same treatment.

Thousand-grain weights of the CGL and WGL fruits were 21.85 ± 0.17 and 14.73 ± 0.21 g, respectively. The relevant *t*-test indicates that the thousand-grain weight of the CGL fruits significantly differed from that of the WGL fruits (*t* = 59.63, d.f. = 7.59, *P* < 0.001). The fruits of the CGL become heavier than those of these wild relatives.

## Discussion

Buoyancy and germination ability of the fruits during and after seawater immersion are adaptations of coastal specialists to seawater dispersal (Nilsson *et al.*, 2010; Yang *et al.*, 2012; Hoyle *et al.*, 2015; Lin *et al.*, 2016). Without these properties, *G. littoralis* cannot disperse and persist along supratidal zones of coasts and will be eliminated or suppressed by surging seawater, with the fruits sinking to seabed or unable to germinate (Yang *et al.*, 2012). Compared with those of the WGL, fruits of the CGL have reduced buoyancy and germination potential. These declines are functional deviations from the WGL, and suggest that the CGL are discarding adaptations to seawater dispersal (Glison *et al.*, 2015; McAssey *et al.*, 2016). However, fruits of the CGL became heavier, showing larger thousand-grain weights. Thus, these declines in fruit buoyancy and germination potential could hardly be attributed to weight changes of the fruits.

The decline of buoyancy in CGL fruits is believed to result from the absence of seawater-dominated selection. The WGL fruits maintain high buoyancy presumably because of continual seawater selection (Nilsson *et al.*, 2010; Yang *et al.*, 2012, 2017). Repeated seawater surge along coastlines is a selective factor to eliminate the fruits that are not adept at keeping afloat during inundation or immersion, thus, enforces the WGL fruits to develop the buoyancy trait (Yang *et al.*, 2012; Guja *et al.*, 2014; Vazačová and Münzbergová, 2014). In contrast, the CGL growing inland were always cultivated by the locals, and, with the cultivation, their fruits were no longer selected by seawater inundation. As a result, fruits of lower buoyancy could not be eliminated, but were saved indifferently for sowing in late years. In this way, the domestication induced the tendency for buoyancy reduction, which has now developed generation after generation for hundreds of years.

The decline of germination potential in CGL fruits might also be related to the lack of seawater inundation. Since the domestication began, the locals selected CGL fruits every year for range-limited sowing, and the CGL could not encounter seawater inundation again. They were completely deprived of the normal process of dispersal, thus, their genes could not circulate freely among broad open populations (DiFazio *et al.*, 2012; Rubio De Casas *et al.*, 2015; Lamont *et al.*, 2016). This dispersal failure, therefore, may have caused serious intra-specific segregation and a series of functional declines in the CGL, and the observed decline of germination potential is one exhibition of the declines (Nathan and Muller-Landau, 2000; Usinowicz, 2015).

The CGL cannot disperse so efficiently as the WGL and have been completely absolved from seawater inundation (Yang *et al.*, 2012). They are governed by abnormal dispersal and functional declines. The domestication over several centuries has made so significant a decline that even though fruits of the CGL were not immersed in seawater, their germination potential was still terribly low. If this decline continues as before, final expiration or extinction may well come true for these CGL (Finch-Savage and Bassel, 2015). Now that humans cannot provide them with required seawater inundation and normal dispersal, human domestication and cultivation cannot be sufficient to conserve *G. littoralis*.

With increasing coastal tourism and exploitation, *G. littoralis* have become endangered (Kerbiriou *et al.*, 2008; Yang and Zhang, 2010; Balestri *et al.*, 2017). *Glehnialittoralis* are observed to persist well only along sandy coasts, where surging seawater acts as a natural and efficient vector to ensure normal population dispersal (Yang *et al.*, 2017). Riparian and other inland areas cannot meet this requirement of *G. littoralis*. On account of this, we suggest that temperate sandy coasts still with WGL around the North Pacific Ocean should be officially designated as nature reserves for conserving this species. If so, this species can be timely rescued and conserved as persistent resources to back up human domestication and cultivation.
